# Incidence of rubella in a state in North-western Nigeria: a call for action

**DOI:** 10.11604/pamj.2016.25.49.10003

**Published:** 2016-09-29

**Authors:** Semeeh Akinwale Omoleke, Henry Chukwuebuka Udenenwu

**Affiliations:** 1 Immunisation, Vaccines and Emergencies, World Health Organisation, Nigeria; 2 Immuisation and Disease Control Unit, State Primary Health, Care Development Agency, Kebbi State, Nigeria

**Keywords:** Rubella, febrile-rash-illnesses, surveillance, Kebbi State, Nigeria, WHO, MMR/RCV vaccine

## Abstract

**Introduction:**

Rubella cases are often under-reported, especially in many developing countries, owing to inadequate attention and weak funding of elimination strategies, despite being an epidemic-prone disease. Based on available data, this paper, therefore, seeks to bring the attention of public health practitioners, researchers and policy makers to threats of rubella in our environment, and also recommend measures to mitigate the threats.

**Methods:**

The authors conducted a retrospective cross-sectional study in which the laboratory results of febrile-rash-illness cases in Kebbi State, Northwest Nigeria, from January 1, 2014 to December 31, 2015 were analysed, using descriptive statistics and chi-square test. We obtained the data set through the routine Integrated Disease Surveillance System and Response being conducted in Nigeria.

**Results:**

A total of 413 febrile-rash-illness cases were reported and investigated in Kebbi State from 2014 to 2015, 5 (3.5%) tested positive for rubella IgM in 2014 while 7(2.6%) tested positive in 2015. There was no statistically significant difference in the incidence of rubella between 2014 and 2015 (p> 0.05). Rubella infection was mainly found in children less than 5 years of age with peak incidence period during the hot season (between February and April). There was no significant sex bias in this study. However, our practice experiences in this environment suggest a systematic under-reporting and under-diagnosis of febrile- rash-illnesses.

**Conclusion:**

There was no statistically significant difference in the incidence of rubella in children in our setting for the 2-years studied. However, there is a potential for increase in the transmission of the disease due to non-availability of routine childhood vaccination against rubella and the systematic under-reporting of suspected cases and weak laboratory support. In order to better appreciate the burden of rubella infection, there may be a need to undertake a prevalence survey, and simultaneously, strengthening case-based surveillance in Northwestern Nigeria. Further, WHO should support national government in accelerating the introduction of rubella-containing vaccine to stem the potential spread of this infectious disease.

## Introduction

Rubella has been considered as a disease of public health importance because of its teratogenic effect on foetus, usually when a pregnant mother gets infected within the first trimester of pregnancy or just before conception [1–3]. A woman infected immediately before conception or within first trimester of pregnancy has 90% chance of her foetus being infected with rubella. The disastrous outcomes of rubella in pregnancy have been well documented in the literature [1–3]. Rubella is an acute, contagious, often mild viral infection usually affecting vulnerable children and young adults. Asides the congenital infection, rubella is a mild illness which often occurs during childhood [3]. Rubella is transmitted through airborne droplets when infected person sneezes or coughs, similar to measles, and 20-50% of all rubella infections occur without a rash or are subclinical [2, 3]. The pattern of rubella outbreak is seasonal, with epidemics occurring every 5-9 years [4]. This is also in congruence with data from The Gambia where epidemics were serologically diagnosed during 1963-1964 and 1973-1974 [5]. Moreover, the prevalence and periodicity of rubella epidemics varies both in the developed and developing countries of the world. Countries with increased rates of vulnerability to rubella infection among women of child-bearing age have been found to have the highest risk of Congenital Rubella Syndrome. A study analyzed regional measles case-based surveillance data collected and reported by 40 countries in the World Health Organisation (WHO) African region for period ranging from 2002-2009 [6]. The study revealed that the majority (95%) of rubella cases reported had occurred in children up to 14 years of age while 6-16% of women of child-bearing age (15 to 49 years) in the region are susceptible to rubella. The majority (63%) of the cases were from rural settings while 37% were from urban settings. A recent study from Tanzania revealed similar findings- age characteristics in children (commoner among under-fives) and significantly higher in rural settings [7]. Prior to the introduction of rubella vaccine, the incidence of CRS varied from 0.1-0.2/1000 live births during endemic periods, and 0.8-4/1000 live births during rubella epidemics [8, 9]. These are essentially studies done in developed countries but little data are available in this part of the world-developing countries. Projections indicate that the burden of CRS in regions that had not started using rubella-containing vaccines (RCVs) by 2008 may be increased. Credence to this, in 1996, approximately 22,000 children with CRS were born in Africa (95% confidence interval, 6127-51 472), and approximately 46 000 (95% confidence interval 1016-168 910) and 12 634 (95% confidence interval 1545-21 396) children were born with CRS in the South-East Asia and the Western Pacific Regions, respectively [8]. Sadly, only small proportion of countries in these regions had introduced RCVs by 2008, therefore, the burden of CRS in these areas is likely to be similar to that projected for 1996. However, there had been reduced incidences of CRS in regions that had attained increased coverage with rubella vaccine during 1996-2008 [8].

In another vein, one of the few studies done in Nigeria, precisely, in Abia State, southeastern Nigeria showed an incidence of new rubella infection of 6.81/1,000,000 population in 2007 which decreased to 2.28/1,000,000 in 2009, but soared to 6.34/1,000,000 in 2011[10]. According to the study, the reason for the decrease in 2009 was not clear since there is no routine rubella vaccination in Nigeria. The authors argued that the decrease could be due to inadequate rubella surveillance activity [10]. Eighty six percent of the new rubella cases occurred in children who were younger than 15 years old. The above result could be compared to 94.7% of IgM positive cases occurring in children younger than 15 years old in Ethiopia [11]. Further, the results of few surveys done in Nigeria among (unimmunized) pregnant and women of child-bearing age to determine the prevalence of rubella showed that between 53% and 77% of the women had rubella, based on the presence of rubella IgG antibodies [12, 13]. The reported prevalence will likely continue to increase as a result of non-introduction of childhood vaccination against the disease and little or no attention being paid towards improving surveillance for CRS and rubella infections. However, the role of vaccination against rubella in reducing the incidence of rubella and congenital rubella syndrome has been demonstrated in most developed countries and a few developing countries [2, 14]. Sadly, the most populous country in Africa- Nigeria, with potentially high case burden of rubella has dearth of data on the prevalence or incidence of rubella infection and is yet to introduce rubella into routine vaccination schedule for under-one or even under-five population. In addition, there is no recent published report on rubella known to the authors from any of northwestern states in Nigeria. Therefore, this paper seeks to bridge these gaps by providing a brief appraisal of rubella infection in a northwestern state over a period of two consecutive years using the case-based surveillance data. This paper also seeks to add to the clamor for the introduction of RCV into routine immunization schedule as well as strengthening of surveillance for febrile-rash illnesses (essentially measles and rubella) in Nigeria.

## Methods

**Study Design:** it is a retrospective cross-sectional study in which all the investigated febrile-rash-illness cases in Kebbi State, Northwest Nigeria, from January 1, 2014 to December 31, 2015 (2years period) were captured through the Integrated Disease Surveillance System and Response.

**Data:** data were collected from the existing 124 disease surveillance focal sites spread across the state. The focal sites are part of the conventional surveillance system domiciled at designated health facilities. These focal sites cut across all the tiers of health care service delivery in the state. Each focal site is manned by a trained focal person who is expected to report *febrile rash* (suspected measles /rubella) cases to a local government area disease surveillance and notification officer (LGA DSNO). The DSNO investigates the cases by collecting blood samples accompanied with necessary documentation. At the health facility level, a suspected case of measles/rubella is said to be 'any person who presents with fever and maculo-papular rashes and any of the following symptoms- cough, coryza and conjunctivitis'. Alternatively, it could be defined as 'any person in whom a clinician suspects measles'. At the community level, it is defined as 'any person who presents with fever and rashes'. There exists an alternative reporting system- use of community informants who are expected to report suspected cases to the focal persons, however, in few instances, notifications are made directly to LGA DSNOs. The DSNOs conduct investigation usually within 48hours of notification. A suspected case of febrile-rash-illness could still be investigated up to 30days from the date of onset of rash. The sera/blood samples collected are sent to the regional measles laboratory where the samples are serologically tested for measles and rubella immunoglobulin M (IgM) antibodies. This zonal laboratory operates according to World Health Organization protocols and standards. Results of laboratory analysis are shared with WHO and the state authorities periodically. However, the authors do not have information regarding the sensitivity and specificity of laboratory technique used in the WHO-accredited laboratory. We exported the laboratory results from access database into excel sheet for statistical analyses. The datasets captured information about the name, age, sex, town/neighbourhood, vaccination status, date of onset and presence of IgM antibodies for rubella (positive or negative). The confidentiality of the data is being maintained by the State Ministry of Health and the World Health Organisation.

**Ethical Consideration:** We made use of secondary data generated from routine case-based surveillance as part of Integrated Disease Surveillance System and Response being practised in the African region- Nigeria inclusive. The authors did not have contact with any of the human subjects as the designated officers in the local government area, i.e., the Disease Surveillance and Notification Officers are saddled with the responsibility of coordinating clinical sampling. In some circumstances, where health workers have been well sensitized, samples are often collected for the DSNOs while he completes the required documentation that will accompany blood sample or serum to the laboratory for confirmation. However, parents of children that were investigated were duly informed of the purpose of clinical sampling (blood sample collection) and verbal consents were taken before samples collection. The World Health Organisation encourages the surveillance officers to also provide feedbacks to parents when the results of laboratory testing are made available from the zonal laboratory. Finally, the authors are aware of the rights of the human subjects and are guided by the World Medical Association Declaration of Helsinki [15].

**Data Analysis:** Descriptive statistical analyses were performed in terms of person (sex and age group) and time. Relative risks were also conducted using statistical software [16]. Categorical variables were presented as proportions and analysed using chi-square to observe the differences among the various groups. The results are summarized in **Table 1**.

**Table 1:** Shows the distribution of suspected and confirmed cases of rubella by year, age, sex and months of disease onset as well as relative risk and test of proportion

Year of Onset	Suspected Febrile Rash Cases Reported	No of positive Rubella IgM	% IgM positive	Relative Risk (95% CI)	P-value
2014	144	5	3.5%		
2015	269	7	2.6%		
**Total**	**413**	**12**			
**Sex**					
Female	187	6	50.0%	1.209 (0.396-3.690)	0.426
Male	226	6	50.0%		
**Age**					
< 5	267	7	58.3%	0.472 (0.154- 1.450)	0.739
5 – 9	90	5	41.7%		
10 – 14	31	0	0%		
> 15	23	0	0%		
**Month of Onset**					
February		2	16.7%		0.190
March		6	50.0%		
April		4	33.3%		

## Results

Of the 413 febrile rash cases reported and investigated in 2014-2015, 5 (3.5%) tested positive for rubella IgM in 2014 while 7 (2.6%) were positive in 2015 (Table 1). We conducted test of proportion using chi-square to ascertain if there is no significant difference (p- value is 0.426) in the proportion of positive cases between the 2014 and 2015 (Table 1). Our field experiences suggest a systematic under-reporting and under-diagnosis of febrile-rash-illnesses. Hence, these results could likely be an under-estimate of the disease burden. Over the 2 years review, cumulative result showed that 50% of positive cases were females, which is not statistically significant (p-value = 0.739). The results also showed that more rubella infections were reported among under-5 years children (58.3%), followed by children in the age group 5-9 years (41.7%) while none was reported among ≥10years. Further, when the relative risk is compared between two age groups of less than 5 years and 5-9years, there was no significant difference in risk between the two age groups (RR- 0.472; CI= 0.154- 1.45) (Table 1). The result also showed that within the years under review (2014 to 2015), there was low incidence of rubella 2 (16.7%) in the month of February, which increased to 6 (50.0%) in March, and reduced to 4 (33.3%) in April (Table 1).

## Discussion

In Nigeria, vaccination against rubella is not conducted in the various health facilities undertaking routine immunization across the country [12], as the vaccine is yet to be formally introduced into the national routine immunization schedule for children. This study explores the occurrence of rubella in a northwestern state of Nigeria with the aim of understanding the epidemiology of rubella in this environment and to add to the clamor for the introduction of RCV into routine immunization schedule as well as strengthening of surveillance for febrile-rash illnesses (essentially measles and rubella) in Nigeria. Of the 413 febrile rash cases reported and investigated in 2014 and 2015, 12 (2.9%) were positive for rubella IgM antibodies. This is small compared to 10.7% of reported febrile cases that tested positive for rubella IgM in Aba, Southeastern Nigeria [10], which is similar to 12% of reported cases in Ethiopia that tested positive for rubella IgM antibodies respectively [11].

A recent study conducted in Tanzania also reported a seroprevalence of 10.9% for rubella IgM antibodies among under-five children [7]. The reason for the small number of positive cases as observed in our study could be attributed to the fact that majority of the analyzed laboratory results were still pending as at the time of conducting the data analysis. Secondly, our study only captured a proportion of symptomatic cases as certain unknown proportion of symptomatic cases might not have been captured by surveillance system, asides the fact some cases of rubella could be asymptomatic. Hence, our finding may be an under-reporting of the incidence of rubella. The challenge of non-processing of samples, and ultimately, non-availability of laboratory results is one of the challenges confronting case-based surveillance for febrile-rash illness (measles & rubella) in Nigeria. Hence, this underscores the need for a prevalence survey to accurately measure disease burden prior to the introduction of vaccination, and strengthening of clinical and laboratory surveillance for rubella. In our study, there was no difference in the positive IgM rubella cases between male and female as both groups had 50% each. This result varies with the studies conducted in other parts of Nigeria, which shows that females had increased incidence of rubella than the males. For example, a study conducted in Jos, North Central Nigeria showed that 33% were males and 67% were females [17]. Similarly, Ume and Onye reported 41% rubella cases among males and 59% in females [10]. In Ethiopia, 54% of IgM positive cases were females higher than 46% reported among the males [11]. However, disaggregating the result of this present study, 2015 data alone showed that rubella positive cases were higher in females (57.1%) than in males (42.9%) than it was in 2014 which is in tandem with the findings of previous studies as mentioned above [10, 11, 17]. There were more reported and investigated cases of rubella that tested positive 7(58.3%) in 2015 than 5 (41.7%) in 2014 in this present study. This could be attributed to improved awareness in 2015, especially among health workers and DSNOs, following sensitization meetings supported by the WHO team in the state. As part of their routine surveillance activities, the DSNOs are expected to conduct active case search at high, medium and low priority sites, including sensitization of health workers on febrile rash reporting as well as other public health diseases of interest.

Rubella incidence occurred within a span of three months as shown in this present study. Of the 413 suspected febrile rash cases reported within the 2 years, all the 12 positive rubella IgM (confirmed) cases were reported in February, March and April. Of the 12 positive rubella IgM cases confirmed, the highest 6 (50%) occurred in the month of March compared to 2(16.7%) in February and 4(33.3%) in April respectively. This finding suggests a possible seasonality in the incidence of rubella infection in the north western part of the country with peak incidence in the month of March. Our finding is similar to the findings of a study in Southwestern Nigeria which observed a seasonality of rubella cases with an increase in the number of cases in the first quarter (January-April) and last quarter (September-December) [18]. It is similar to the findings from Ethiopia which observed increase in the number of rubella cases in February, with a major peak between April and May before cases declined to the lowest level in August [11]. The rate of rubella IgM positivity in children less than 5 years in this study is 58.3% which is higher compared to rubella IgM positive rate of 10.8% among children of the same age group in Aba, southeastern Nigeria [10]. However, the result of this present study is similar to the study conducted in Jos, north central Nigeria in which children between 0-10 years had rubella IgM positive rate of 45.2% [17]. In the study by Ume and Onyi [10], 86% of rubella positive cases were reported in children younger than 15 years. This is also similar to the findings of Mitiku and colleagues [11] that reported 94.7% of IgM positive cases in children younger than 15 years old in Ethiopia. Like previous reports, our study result also concurs with the observations of WHO that rubella is a disease that mainly affects children [2]. The zero occurrence of rubella cases among children older than 10 years as recorded in our study might not be unconnected with the fact that susceptible population decreases with increasing age. This could further be explained with the fact that a greater percentage of the population would have been infected and developed immunity before adulthood without being symptomatic.

**Limitations of the study:** The study made use of relatively small sample size of analysed blood specimen yielding relatively small numbers of positive rubella IgM cases. These results may not likely be the true representation of the burden of rubella in Kebbi State population. However, this shortcoming is one of the strengths or revelations from this report, i.e., weak laboratory surveillance as some samples were not tested due to logistic challenges at the designated zonal laboratory. Further, field experiences suggest under-reporting of febrile-rash illnesses, hence, the data presented may not be representative of the burden of disease. However, we have been able to demonstrate the presence of rubella with a potential of disease transmission from children to adults due to the absence rubella-containing vaccines. Lastly, the small sample size might have accounted for the absence of sex disparity observed in this present study.

## Conclusion

The study provides a clue regarding the burden of acute rubella infection in this environment, despite the aforementioned study limitations and challenges of case-based surveillance for febrile-rash illnesses. Hence, the need for governments at all levels to take lead towards strengthening case-based surveillance for febrile rashes, especially laboratory surveillance, and hastening the introduction of RCV to prevent and control acute rubella infection and congenital rubella syndrome. **Recommendations:** Our practice experiences suggest that the major challenges to rubella surveillance in Nigeria, include lack of support and funding from the government leading to shortage in operational facilities such as laboratory reagents. In addition, the role of WHO in providing funding support for surveillance activities in Nigeria which should be complementary has become substitutive and may not sustainable. Hence, Rubella surveillance in Nigeria should be improved vis a vis conducting laboratory diagnosis for all reported febrile rash cases, at least in all state public health laboratories and tertiary health facilities. Government should intensify support for surveillance with robust funding to adequately fill operational gaps such as human resources and required logistics. There may be need to conduct a seroprevalence survey to accurately measure the disease burden in northwestern Nigeria. Rubella-containing vaccine should be introduced into the national routine immunization schedule to avert the potential threats of rubella epidemics (rubella in children and CRS) in the country.

### What is known about this topic

- Paucity of data on congenital rubella syndrome and rubella infection in children in Africa, including Nigeria;
- The seasonality of occurrence of rubella infection in Nigeria which is similar with study from East Africa (Ethiopia);
- The positive impact of introduction of rubella-containing vaccines in developed countries were of note as there were sharp declines in incidences of congenital rubella infections and other complications associated with rubella infection.

### What this study adds

- We did not find a statistically significant difference in the incidence of acute rubella infections between 2014 and 2015, however, this study is an eye-opener to the fact that there may be need for a more robust survey to accurately determine the burden of the disease, given the weakness of existing surveillance system (under-reporting and diagnostic challenge) and presence of asymptomatic or sub-clinical cases of rubella;
- The need to improve laboratory surveillance emerged in this paper based on our local experience between 2014 and 2015, as laboratory results were not readily available after clinical sampling. There is need to accelerate the introduction of rubella-containing vaccine to prevent the negative impacts of rubella infections and this should be complemented with robust routine surveillance for febrile rash illnesses;
- Government should take pragmatic steps in complementing the current effort by the World Health Organisation towards ensuring constant availability of laboratory reagents and related logistics for laboratory confirmation of suspected cases (laboratory surveillance).
